# Addressing electronic and dynamical evolution of molecules and molecular clusters: DFTB simulations of energy relaxation in polycyclic aromatic hydrocarbons

**DOI:** 10.1039/d3cp02852f

**Published:** 2023-10-19

**Authors:** Mathias Rapacioli, Maysa Yusef Buey, Fernand Spiegelman

**Affiliations:** a Laboratoire de Chimie et Physique Quantique (LCPQ/FERMI), UMR5626, Université de Toulouse (UPS) and CNRS 118 Route de Narbonne F-31062 Toulouse France mathias.rapacioli@irsamc.ups-tlse.fr

## Abstract

We present a review of the capabilities of the density functional based Tight Binding (DFTB) scheme to address the electronic relaxation and dynamical evolution of molecules and molecular clusters following energy deposition *via* either collision or photoabsorption. The basics and extensions of DFTB for addressing these systems and in particular their electronic states and their dynamical evolution are reviewed. Applications to PAH molecules and clusters, carbonaceous systems of major interest in astrochemical/astrophysical context, are reported. A variety of processes are examined and discussed such as collisional hydrogenation, fast collisional processes and induced electronic and charge dynamics, collision-induced fragmentation, photo-induced fragmentation, relaxation in high electronic states, electronic-to-vibrational energy conversion and statistical *versus* non-statistical fragmentation. This review illustrates how simulations may help to unravel different relaxation mechanisms depending on various factors such as the system size, specific electronic structure or excitation conditions, in close connection with experiments.

## Abstract 2

We present a review of the capabilities of the density functional based Tight Binding (DFTB) scheme to address the electronic relaxation and dynamical evolution of molecules and molecular clusters following energy deposition *via* either collision or photoabsorption.

## Graphical abstract

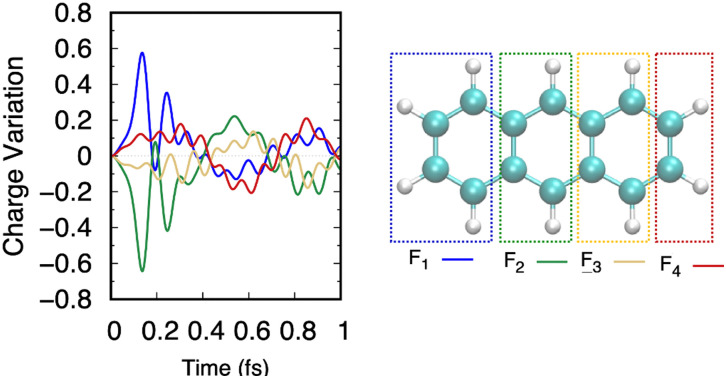

## 1 Introduction

Polycyclic aromatic hydrocarbons (PAHs) are organic compounds with a carbonaceous skeleton made of fused 6-member carbon atom rings terminated by hydrogen atoms at their borders. They are also sometimes called hydrogenated graphene nanoflakes^1^ as they can be regarded as small pieces of a graphene layer. However, the electronic properties of PAHs differ from those of graphene as their finite size induces the opening of an energetic gap between the HOMO and the LUMO. The presence of a delocalised π resonant system makes PAHs particularly stable molecules and usually enforces the structures to be planar. The simple Clar's rule^2–4^ is mostly sufficient to explain the relative stabilities of various PAH structures that differ by ring arrangements.

In an astrophysical context, PAHs have been proposed as carriers of a series of astrophysical infrared features, nowadays known as aromatic infrared bands (AIBs). Initially observed in the interstellar medium (ISM),^5^ these bands appear to be ubiquitous in the universe, as they have been reported in various planetary nebulae, the galactic interstellar medium, star forming regions or external galaxies.^6–8^ The bands located at 3.3, 6.2, 7.7, 8.6, 11.3 and 12.7 μm have been attributed to characteristic C–C and C–H vibrational modes^9,10^ of PAHs in the mid 80's. The broadening of these bands, probably due to the presence of a large number of species whose spectra present slight variations combined with thermal anharmonic effects, makes the identification of a single precise molecule challenging.^11^ However, it is worth mentioning that cyano-naphthalene^12^ has been the first interstellar PAH identified and that benzene and fullerenes (not strictly belonging to the PAH family) have also been identified.^13–16^ The fact that, depending on their environment, AIBs only present slight variations^17^ has been interpreted as an argument either for the hypothesis that PAH growth in space results in a limited number of large and compact PAHs^18^ or for the hypothesis that different mixtures of PAHs, with a large variety of sizes and chemical patterns, would have similar mean spectral signatures.^19^ In addition to free flying PAH molecular units, PAH clusters have been proposed to explain spectral band broadening and the apparition of a continuum contribution underlying the AIB features observed in the most UV protected regions of various astrophysical objects.^20–22^ PAH clusters have also been proposed as contributors to the broad photoluminescence extended red emission (ERE)^22,23^ or diffuse interstellar bands (DIBs).^24^ Given the fact that PAH compounds may contain up to 20% of interstellar carbon,^25^ it is essential to characterise their role in gas heating, their thermal and dynamical relaxation, their chemical evolution, including growth and fragmentation, as well as their catalytic role, in particular regarding molecular hydrogen formation.^26–33^ PAH-based systems can undergo various energy deposition processes. In photodissociation regions (PDRs), *i.e.* molecular clouds where the physics/chemistry is dominated by one or several nearby stars' UV fluxes, they can absorb photons with energies below 13.6 eV, an upper bound resulting from the high density of hydrogen atoms which absorb higher energy photons. In addition to photoexcitation or photoionization, collisions with stellar wind particles also play a significant role in circumstellar environments.^34,35^ Collision shock wave regions as well as cosmic ray irradiation should also be mentioned as sources of high energy deposition in these systems.^36,37^ It is then crucial to decipher the interplay between the energy deposition processes and the subsequent dynamical evolution. Understanding the role of PAHs as possible precursors of fullerenes and carbon nanotubes is a very active research field in the astrophysical context but it is also of interest in nanosciences.^26,38,39^ It should also be noted that PAHs and PAH clusters have been widely investigated in the context of soot formation in flames^40–57^ and that, in such a context, it is also mandatory to understand the evolution of these systems after absorbing energy.

Experimentally, the fragmentation of PAHs resulting from UV irradiation or collision has been investigated evidencing the dominant H, H_2_ and C_2_H_2_ loss channels and characterizing the stability of excited PAHs.^58–70^ Collision- or photon-induced fragmentation has also been characterized in PAH clusters.^71^ These experiments have demonstrated that the resulting mass spectra and identified dissociation channels can be interpreted schematically as belonging to one of two typical scenarios (see reviews^11,72^), namely statistical or non-statistical fragmentation patterns which are determined by the nature of the excitation and the characteristic timescales of energy redistribution. The statistical fragmentation scenario assumes that the deposited energy is redistributed equally over the vibrational modes of the electronic ground state prior to any fragmentation/isomerization. Statistical fragmentation may result from energy deposition in the electronic system, *i.e.* UV photon absorption or collisions with a high energy (several tens of keV) ion for which the electronic stopping power dominates over the nuclear stopping power, followed by efficient internal conversion (IC) toward the electronic ground state^73–76^ and efficient intramolecular vibrational redistribution (IVR). Statistical fragmentation can also correspond to direct excitation of the nuclear vibrational modes. This is the case in IR multi-photon absorption experiments^77–79^ or in low energy collisions.^80^ The non-statistical fragmentation may correspond either to fast dissociation in the electronically excited states or to knockout collisions, *i.e.* when the nuclear stopping power dominates and evaporation of a single atom or small fragment occurs before the absorbed energy redistribution.^72,81–86^ The non-statistical processes may strongly depend on the details of energy deposition (topology of the excited electronic state, location of a collision impact point) while the statistical processes are driven by the amount of deposited energy. Let us finally mention that nowadays, the ultrafast evolution of photoexcited PAHs can also be monitored thanks to the recent advances in femtosecond pump–probe experiments.^87–89^

On a theoretical side, the level of theory chosen to model PAH systems is mostly driven by the computational cost required to simulate such extended systems. At the DFT level, it is possible to compute energies of characteristic PAHs, their isomers and fragments to gain insights into the fragmentation channels. The additional determination of dissociation and isomerization barriers allows the connection of the possible isomerization and dissociation paths^90–93^ and the identification of the dominant ones. Their statistical rate constants can be derived for a given energy or temperature from statistical theories once vibrational frequencies are computed.^94,95^ DFT has also allowed the structural and energetic properties of PAH clusters to be addressed^96–103^ and one should also mention a few studies at the MP2^102–104^ or SAPT^105^ levels. It is worth mentioning that modelling molecular clusters is a challenging task at the DFT level as one should appropriately include dispersion interactions^106^ as well as self-interaction corrections to effectively solve the charge resonance issue in ionic clusters.^107^ Semi-empirical potentials ranging from empirical tight binding schemes to force field approaches allow for millions of single point energy and gradient calculations, opening the route to extensive global exploration of potential energy surfaces. With such potential, molecular dynamics simulations^108–110^ or extensive Monte Carlo explorations^111^ of PES can be performed. This allowed the structural diversities and thermodynamics of hydrogenated-carbonaceous clusters^112–114^ and PAH clusters to be probed^115^ and the competition between the various fragmentation and isomerization channels following energy deposition to be quantified.^116^ The density functional based tight binding (DFTB) approach^117–119^ lies in between semi-empirical and *ab initio* methods. The use of a minimal atomic basis and parameterized integrals allows for a computational cost similar to that of semi-empirical tight binding schemes, while its DFT grounding allows for better transferability and often provides systematic ways of improvements. In particular, DFTB is well suited to describe chemical reactivity at a low computational cost.

In this review, we present the capabilities of the DFTB scheme to simulate the dynamical evolution of molecules and molecular clusters after energy deposition. The present review is restricted to the simulation of PAHs and PAH clusters which are relevant in astrophysical and atmospheric science, but the latter can also be seen as prototypes of molecular systems for the presented simulation techniques. In the next section, we provide an overview of the DFTB method basics and some possible extensions, in particular to address excited states or to deal with resonance processes in molecular clusters, as well as the coupling of the DFTB potential with adiabatic and non-adiabatic dynamical schemes. Section 3 is devoted to the simulation of PAHs and PAH clusters receiving energy from a collision whereas Section 4 addresses the dynamical evolution of such systems receiving energy from photon absorption. Finally, a discussion and perspectives are drawn in the last section.

## 2 Density Functional based Tight Binding (DFTB)

### 2.1 DFTB basics

Tight binding approaches have been developed since the early years of quantum physics and chemistry, leading to the Huckel and extended Huckel models^120–123^ in the chemistry community and tight binding equivalent models in solid states and surface physics.^124–128^ In these schemes, only a subpart of the electronic system is treated explicitly, for instance considering only π electrons in planar systems making use of the Pariser–Parr–Pople^129,130^ approximation or only the valence electrons similarly to the frozen core or pseudopotential approach in *ab initio* schemes.

The energy of the system includes the kinetic energy of the nuclei, a repulsive atomic-pair potential and a so-called electron energy (sometimes called band energy). The latter is obtained by diagonalization of a parameterized Hamiltonian:

1 *H*^TB^*C*_*i*_ = *ε*_*i*_*SC*_*i*_

where *C*_*i*_ is the vector of the coefficients of the molecular orbital (MO) *ϕ*_*i*_ expressed in a minimal atomic base {*φ*_*μ*_} (orthogonal or not): 
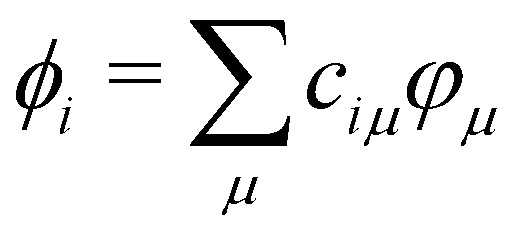
. *H*^*TB*^ and *S* are the Hamiltonian and overlap matrices expressed in this atomic basis. Each matrix element, *H*^TB^_*μν*_ or *S*_*μν*_, is expressed as a one- or two-body term, depending only on the relative positions between the atoms bearing the orbitals *μ* and *ν*. The electronic energy of the system is obtained by summing the energies *ε*_*i*_ for the occupied orbitals *ϕ*_*i*_ weighted by their occupation numbers *n*_*i*_.

Although these schemes have often been considered as empirical schemes based on chemical/physical intuitions, Foulkes and Haydock^131^ demonstrated that a tight binding algebraic expression can be derived from DFT equations by a first order expansion of the energy with respect to electronic density fluctuations. In doing so, these authors established the missing link between *ab initio* approaches and TB methods, thus providing rigorous justification for these empirical approaches developed since Hückel in the 1930s. This work was followed a few years later by the group of G. Seifert and T. Frauenheim^117,118^ who established the Density Functional based Tight Binding (DFTB) method. DFTB is derived from DFT making use of the three main approximations: (i) the expansion of the DFT energy with respect to a reference electronic density as suggested by Foulkes and Haydock^131^ (ii) the use of a minimal valence atomic basis and (iii) the neglect of more than two center integrals. There are several versions of the DFTB scheme, depending on whether the Taylor expansion is performed at first or second order. At the 1st order, the DFTB energy presents an algebraic formulation identical to the previously mentioned TB models, the only difference being the replacement of *H*^*TB*^ in eqn (1) by the matrix *H*^0^ taken as the DFT/KS operator computed with the reference density. The method was refined in 1998 by Elstner *et al.*,^132^ by integrating second order terms in the Taylor expansion. In this latter version, the most widely used so far, the energy expression is2
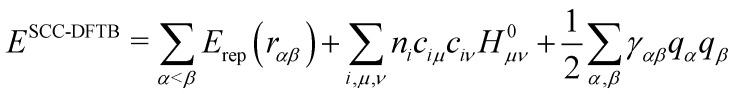
where *E*_rep_(*r*_*αβ*_) is a repulsive contribution between the atoms *α* and *β*. The second term is the band energy and the last term contains the second order corrections expressed as a function of the atomic charges *q*_*α*_ and a *γ*_*αβ*_ matrix. Its diagonal elements are atomic chemical hardnesses (or Hubbard parameters) and the off-diagonal terms describe the 1/*R* coulomb interactions between atomic charges plus an exchange–correlation energy contribution. The second order correction introduces a charge dependency in the operator *H*^1^(*q*) and the new secular equation

3 (*H*^0^ + *H*^1^(*q*))*C*_*i*_ = *ε*_*i*_*SC*_*i*_

must therefore be solved self-consistently as the atomic charges *q*_*α*_ depend on the MOs *c*_*iμ*_ coefficients. This method is also known as self-consistent-charge (SCC) DFTB. Thus, it is interesting to note that second-order terms reintroduce self-consistency, similarly to what is done for the DFT electronic field (self-consistent field). Note that formulations up to third-order terms in the Taylor expansion have been made, introducing Hubbard's term dependency with occupation.^133^

The traditional parameterization recipe involves DFT calculations for atoms and atomic pairs only. Theoretically, the DFT-based parameterization gives DFTB greater transferability than empirical schemes parameterized for specific systems and whose accuracy is questionable away from the parameterization domain. From the *ab initio* perspective, DFTB can be seen as an approximate DFT scheme whose strength lies in its reduced computational cost, at the price of the above approximations. The major difficulty is that the parameters must exist or be developed for all types of atomic pairs present in the system. With respect to traditional TB methods, the strengths of DFTB lie in its DFT grounding, leading to an *ab initio* parameterization recipe and *a priori* greater transferability.

Since the development of DFTB in the mid 90's, many improvements have been proposed. They include the adaptation of DFT improvements to the DFTB framework or improvement of the approximations of DFTB with respect to DFT. Let us cite in a non-exhaustive way: developments aiming at going beyond the Mulliken approximation to map the electronic density,^134,135^ the introduction of dispersion contributions,^136–138^ the unrestricted formulation introducing spin penalty terms,^139^ the extension to periodic conditions allowing for band structure calculations,^140,141^ and the separation schemes to introduce long range HF exchange.^142^ Finally, DFTB can be coupled with lower level methods in a QM/MM scheme to include environmental effects^143–146^ or with higher level theories (like DFT) in a QM/QM' scheme.^147^

An important advance in the description of the mechanisms of electronic relaxation was the access to electronically excited states which was developed using the Linear Response approximation of Time-Dependant DFTB (LR-DFTB),^148^ similar to the Casida equations at the DFT level.^149^ In LR-DFTB, the excitation energies are given as eigenvalues *Ω*_*I*_ of the following matrix equation:4
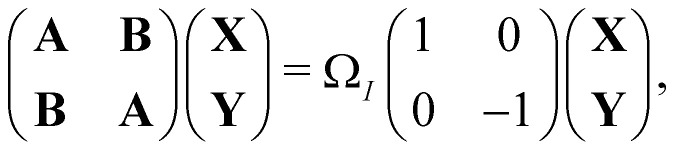
where 
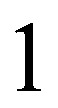
 is the identity matrix. The **A** and **B** matrix elements are given by

5 *A*_*ia*,*jb*_ = (*ε*_*a*_ − *ε*_*i*_)*δ*_*ij*_*δ*_*ab*_ + 2*K*_*ia*,*jb*_;

6 *B*_*ia*,*jb*_ = 2*K*_*ia*,*jb*_;

Indices *i*,*j* and *a*,*b* label the occupied and virtual MOs with energies *ε*_*i*_ and *ε*_*a*_, respectively. Within the DFTB approach, the coupling matrix elements *K*_*ia*,*jb*_ are usually calculated from the DFTB Mulliken transition dipoles.^148^

### 2.2 Extensions of DFTB for molecular clusters

The interested reader can find details about DFTB and these extensions in several reviews^140,150–152^ and we detail now only those which are essential for the specific treatment of molecular clusters. The standard SCC-DFTB often fails at modelling molecular clusters and specific corrections must be considered. Neutral molecular cluster structures result from a delicate competition between Pauli repulsion, Coulomb interactions resulting from the charge fluctuations of the fragments, polarization forces and dispersion interactions. The dispersion interaction is almost absent in DFTB, but several empirical corrections have been implemented^136,137^ relying on a dispersion term C_6_/R^6^ associated with a short-distance screening function. The description of the intermolecular Coulomb potential from atomic charges, calculated using the Mulliken approach, is another source of error. The polarity of the bonds is not well accounted for electron density distributions on atoms. This can be corrected following the approach developed by the Truhlar group (charge model 3, CM3^153,154^) correcting Mulliken charges:7
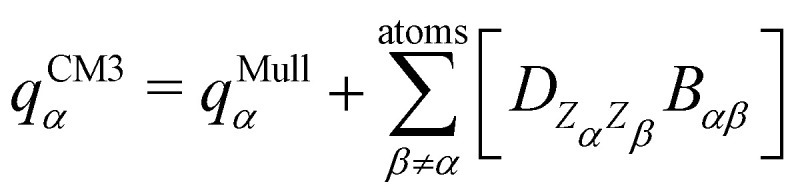
where *B*_*αβ*_ is the Mayer bond order^155^ computed from the density matrix and 
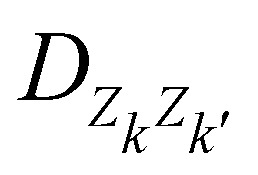
 is an empirical parameter per type of pair of atoms. The combination of CM3 charges with dispersion correction was validated by its ability to recover the fine competitions between T or sandwich structures which are important for benzene and small PAHs,^134^ in agreement with reference calculations (CCSD(T),^156^ SAPT^157,158^). As an alternative, the weighted Mulliken charge^159^ approach introduces a bias in the sharing of the interatomic density matrix elements8
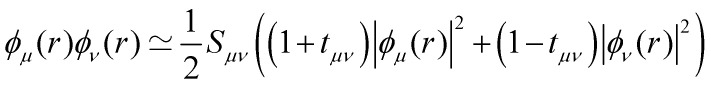
where *t*_*μν*_ is an empirical parameter between −1 and 1 (Mulliken charges are recovered for *t*_*μν*_ = 0). This approach called WMull (Weighted Mulliken) does not increase the computational cost in contrast to the CM3 scheme. The CM3 and WMull corrections give very similar results.^159–161^

Molecular cation clusters provide specific challenging cases. Actually, the description of the charge resonance between the different units is hindered at the DFTB level. The first problem is the well-known long-range self-interaction error, which already exists at the DFT level with standard functionals. It becomes stringent in case of strongly multiconfigurational nature of the exact wavefunction at dissociation,^107^ a defect that standard functionals are unable to correct. A related problem observed in DFT or DFTB is dissociation into fragments bearing fractional numbers of electrons.^162–164^ In the case of a cationic dimer, this results in an irrelevant behaviour of the dissociation potential curve presenting an artificial Coulomb repulsion between two half-charge units towards dissociation and an overestimation of the binding energies. At the DFT level, range separated approaches^165–167^ aim to address the lack of long-range exchange–correlation. Alternatively, Wu and Van Voohris^168^ solved the charge delocalisation issue by combining DFT and configuration interaction (CI) schemes. This method was adapted in the DFTB-CI scheme^169,170^ to restore at a low computational cost the multi-configurational nature of the wave function within the DFTB framework. Taking the case of a cation dimer (AB)^+^ as an example, the wave function can be decomposed on a basis of configurations where the charge is localized on either A or B: |*Ψ*_(AB)^+^_〉 = *a*|*Ψ*_A^+^_〉 + *b*|*Ψ*_B^+^_〉. The coefficients of the wavefunction and the energy of the system *E*_(AB)^+^_ are obtained from the diagonalisation of a small matrix expressed in a valence bond configurational basis:9
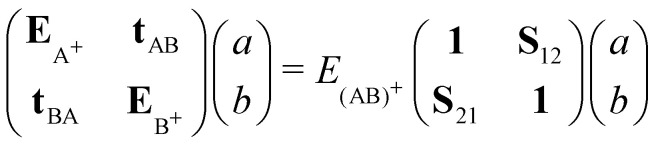
The matrix elements (*E*,*t*,*S*) drive the coupling between the two DFTB charge-constrained configurations and the scheme can be generalised to a cluster containing several molecular sub-units A, B, C,…. Charge-constrained electronic calculations^169–171^ are performed to obtain a basis of charge-localized configurations for each of the M = A, B, C,… unit with a modified DFTB operator involving a Lagrangian constraint:

10 (*H*^0^ + *H*^1^(*q*) + *λ*_M_*P*_M_)*C*_*i*_ = *ε*_*i*_*SC*_*i*_

where *P*_M_ is a density projector on sub-unit M and *λ*_M_ is a Lagrangian parameter, determined to enforce the charge to be localised on sub-unit M. This defines the DFTB energies used as the diagonal elements **E**_M^+^_ in eqn (9) and the MOs are used to build the configurations *ψ*_M^+^_ as a single Slater determinant. These configurations *ψ*_M^+^_ are used to compute the overlap and hopping terms as in eqn (9). In this scheme, the dynamical correlation is treated using constrained DFTB and the static correlation associated with the charge resonance is treated through their interaction at the CI level. No DFTB calculation is performed for a system with fractional charges on the sub-fragments and therefore exhibiting a self-interaction artifact.

The electronically excited states of molecular clusters also involve resonance processes between the different units. Indeed, the cluster's excited states can be expressed as linear combinations of excitations localized on the different sub-units. In order to treat charge and excitation resonance in the same model, the DFTB-CI model has been extended leading to the DFTB-EXCI model.^172^ Configurations corresponding to localized excitations on the different units are added to the CI matrix (eqn (9)). In the case of the dimer, the new configurations are {*ψ*_A+*B_,*ψ*_AB+*_,*ψ*_A+B*_,…*ψ*_A+**B_} where symbol * (resp. **) indicates a first (resp. second) localized excitation on one of the two sub-fragments. The diagonalisation of the extended CI matrix (eqn (9)) restores the delocalization of the excitations and the oscillator strengths can be computed from the transition dipoles.

**Fig. 1:**
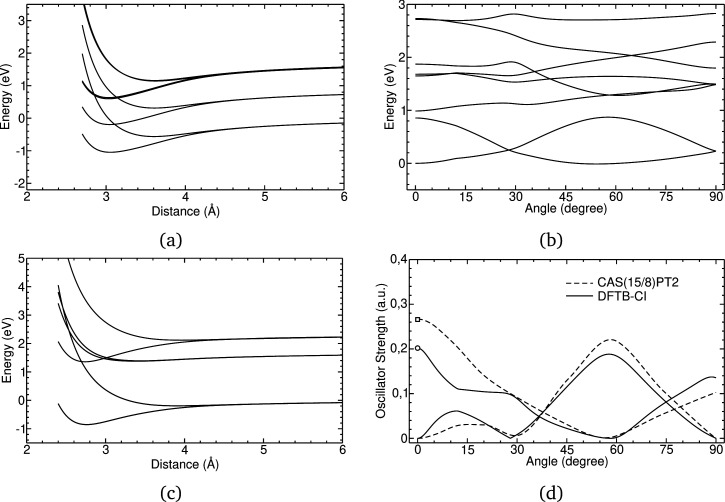
DFTB-EXCI energetic profiles for ground and excited states of cationic dimers of pyrene (a) and (b) and benzene (c) in a superimposed approach path (a) and (c) or following a twisting mode (b). Oscillator strengths for the two first excitations along this path are also reported together with CASPT2 results (d). Adapted from ref. 172 with permission from the PCCP Owner Societies. Copyright 2016.

In Fig. 1, the energetic profiles of benzene and pyrene cation dimer excited states are shown in eclipsed or superimposed plane parallel approaches. In the case of benzene (Fig. 1(c)), the six curves converge at long distance toward three dissociation energies corresponding respectively to the energies of neutral benzene plus the energy of a benzene cation, the latter being in its (i) electronic ground state (hole in π HOMO) (ii) 1st excited state (hole in *σ* HOMO−1) or (iii) 2nd excited state (hole in π HOMO−2). These states split at short distances, due to resonance processes. The splitting intensity depends on the overlap between the hole accepting orbitals and is therefore larger between configurations corresponding to hole resonances between π orbitals than between *σ* orbitals. In the case of the pyrene cation, all of the first excited states considered correspond to holes in π orbitals, leading to similar splittings (Fig. 1(a)). Whereas the states only couple two by two in the eclipsed superimposed approach, symmetry breaking allows mixing between them as shown in Fig. 1(c) corresponding to the twisting of one pyrene *vs* the other. The DFTB-EXCI approach was validated (electronic structure and oscillator strength – Fig. 1(d)) on the basis of comparisons with *ab initio* (CASPT2 calculations). The computation of the latter took several weeks to complete, whereas the DFTB-EXCI results were obtained in a few minutes.

### 2.3 Coupling DFTB with dynamics

DFTB has been routinely used to simulate the dynamical evolution of molecular systems in their electronic ground state. Making use of the Born Oppenheimer approximation, the nuclei are propagated classically following Newton's equations of motion, the energy and its gradients being computed on the fly. A variety of thermostats/barostats can be used to remain in the desired ensemble. Such MD simulations allow the simulation of collisional processes and further fragmentation or isomerization of molecular systems (see Sections 3.1, 3.2.2, 3.3, 4.2). The computational efficiency of DFTB/BOMD can be improved within the Car–Parrinello approach^173^ or by introducing biases in the dynamics to increase the ergodicity like for example in metadynamics.^174–176^

In the case of electronic excitation, several strategies can be followed. In the simulation of ultrafast processes where the nuclei can be considered at fixed positions, only the electronic dynamics need to be described. This is the domain of application of the so-called Real-Time Time-Dependant DFTB scheme (RT-DFTB) equivalent to RT-TDDFT^177^ where the electronic system is propagated with the time dependent Schrödinger equation making use of the DFTB operator.^178^ In practice, it is often the density matrix which is propagated using the Liouville-von Neumann equation11
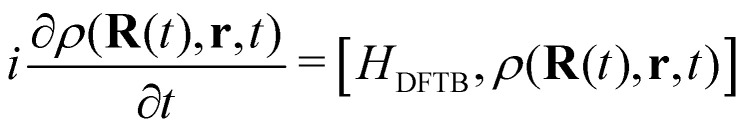


The proper treatment of electron–nuclear coupled dynamics would require the propagation of electron–nuclear wavepackets which is at present out of reach for systems with tens/hundreds of atoms as is the case of PAH and PAH clusters. However these processes can be addressed by so-called mixed classical-quantum dynamics schemes which involve various approximations with respect to wave packet dynamics.^179^ The two most popular schemes, namely mean-field and surface hopping, have been adapted to the DFTB scheme. In the former, also known as Erhenfest dynamics, the electronic system is propagated according to the time-dependent DFTB equation, possibly eqn (11), and the nuclei are propagated classically in a mean time-dependent electronic potential12
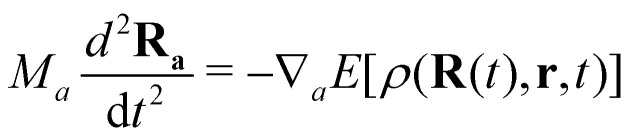
where *ρ*(**R**(*t*),**r**,*t*) is now the time-dependent electronic density. Niehaus *et al.* derived the time-dependant DFTB equations from an extended Lagrangian including the DFTB second order contributions.^180^

Alternatively, in the Trajectory Surface Hopping (TSH) scheme, mostly performed following Tully's fewest switch approach,^181,182^ the nuclei are propagated classically on a given electronic excited state PES and the non-adiabatic couplings13
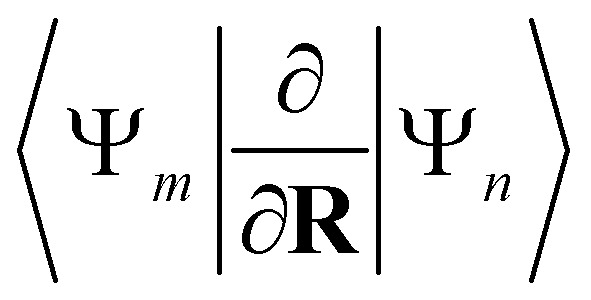
are involved in the probabilities of hopping between states *Ψ*_*m*_ and *Ψ*_*n*_. This approach requires the computation of electronic excited states achieved *via* LR-TDDFTB and their gradients. The derivation of the gradients relies on the so-called *Z*-vector method initially developed for LR-TDDFT^183,184^ before being extended to LR-TDDFTB.^185,186^ Several implementations of DFTB surface hopping dynamics have been reported^187–191^ Let us finally mention another original non-adiabatic MD scheme derived in the context of electronic transport.^192,193^

To conclude this Method section, we mention that DFTB calculations can nowadays be performed with several codes like DFTB+,^194^ deMonNano,^195^ ADF,^196^ Amber,^197^ Gromacs,^198^ Gaussian,^199^ DFTBaby,^187^ and CP2K.^200^ Most of the applications to PAHs and PAH clusters reported in the remaining part of this review have been performed using the deMonNano code^195^ which contains most of the previously presented DFTB extensions and MD couplings.

## 3 Collision induced dynamics

In this section, we address PAH fragmentation and PAH cluster fragmentation/dissociation induced by collision, the choice of the specific systems and collision conditions being mostly driven by the available experiments.

### 3.1 Hydrogenation of PAHs

**Fig. 2:**
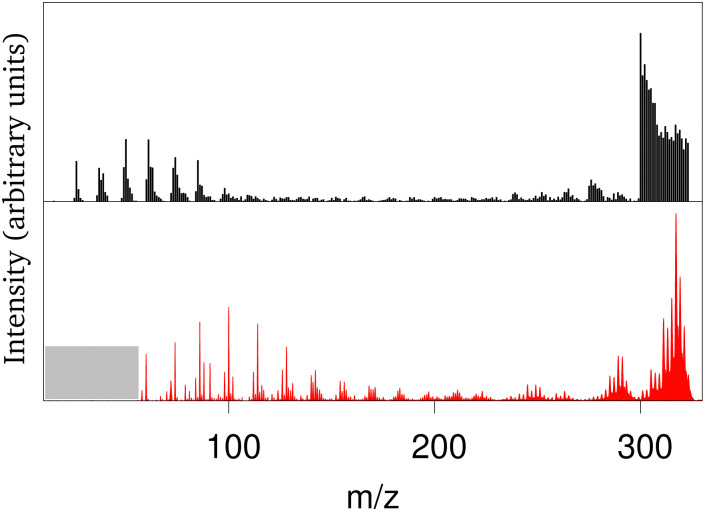
Experimental (black) and calculated (red) mass spectrum in arbitrary units for coronene heated by successive hydrogenations (*m*/*z* = 300: bare coronene; *m*/*z* > 300: hydrogenated coronene; *m*/*z* between 275 and 285: loss of C_2_H_*n*_ fragments). Adapted from ref. 201 with permission from the PCCP Owner Societies. Copyright 2018.

The collision of atomic hydrogen atoms with a PAH may lead to the formation of chemical bonds, provided the collision energy is high enough to overpass the associated chemical barriers (when existing). In the case of neutral PAHs, the highest barrier corresponds to the first hydrogenation.^202^ From energetic considerations, Cazaux *et al.*^203^ showed that the sequential hydrogenation of a cationic PAH follows a specific structural route. Experimentally, several groups have addressed the stability of hydrogenated PAHs (X-ray experiments at 285 eV or collisions with energies between 20–200 eV) leading to apparent contradictions on the possible hydrogenation induced stabilisation or destabilisation of the PAHs.^204–206^ The evolution of successively hydrogenated PAHs can be followed by recording mass spectra as a function of time, *i.e.* as a function of the PAH exposure to hydrogen atoms.^203^ Direct simulation of the experiment is challenging at the DFTB level due to the difficulty to reproduce hydrogen attachment barriers resulting from intersystem crossings and the fact that de-excitation channels like photo-emission should be taken into account on long timescales. It is however possible to perform MD simulations of PAHs with all possible degrees of hydrogenation at various energies and compute mass spectra associated with different scenarios regarding the competition between heating from the release of the chemical absorption energy and photoemission cooling.^201^ Only a scenario assuming an efficient cooling of the highly hydrogenated species provides fair agreement with experimental results. This was interpreted in light of the Poincaré (also called recurrent) fluorescence process where part of the vibrational energy flows back into the electronic degrees of freedom (reverse internal conversion) and the system relaxes then through electronic fluorescence.^207^ This process is already expected to be important for bare (*i.e.* non-superhydrogenated) PAHs and to compete with vibrational emission, especially at high excitation energies.^208^ Hydrogenating PAHs results in a decrease of the HOMO–LUMO gap which favors the population of excited states increasing the recurrent fluorescence process. With this relaxation scenario, the experiments and theoretical mass spectra present concordant features, namely: (i) the survival of highly hydrogenated species, (ii) a bimodal shape for the mass spectrum with either the loss of C_2_H_*n*_ fragments, either very small fragments (see Fig. 2), (iii) a distribution of small fragments shifted towards the smallest masses when the degree of hydrogenation of the parent PAHs increases, the opposite being observed for the largest fragments. This work also showed that a low level of hydrogenation acts as a PAH protection, as the loss of hydrogen atoms is a channel to evacuate energy while preserving the PAH structure. In contrast, a high degree of hydrogenation destabilizes the π electronic system making easier the loss of carbon containing fragments (*i.e.* breaking of the PAH backbone structure).

### 3.2 Ion-PAH collision

#### 3.2.1 Ultrafast collision induced electronic dynamics

**Fig. 3:**
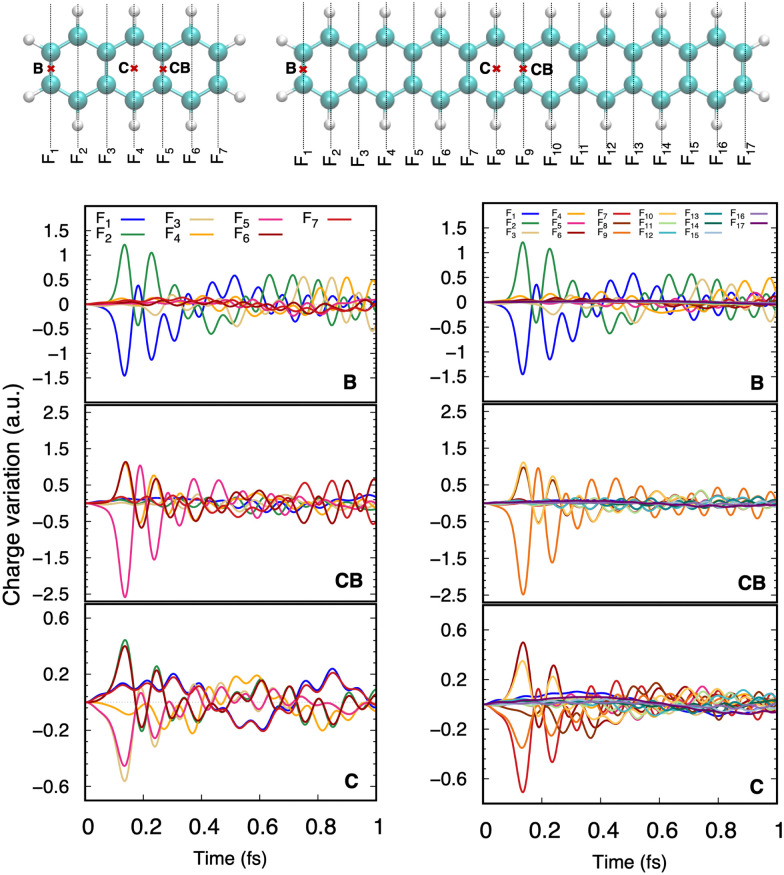
Ultrafast charge evolution for anthracene and octacene after collision with a 100 keV proton impacting the center of a central aromatic ring (C) or a central (CB) or border (B) bond.

In circumstellar environments, PAHs undergo collisions with stellar wind protons and possibly multiply charged atoms. At such high energy (above keV), chemisorption is not possible and, on short timescales, only the electronic cloud reacts to the perturbation. The energy deposition and the induced electronic dynamics can be simulated using RT-TD-DFTB (see Section 2.3). This was performed for anthracene and octacene submitted to proton collision at 100 keV of collision energy. Here, as a preliminary demonstration, the proton trajectory is run perpendicular to the PAH plane and the impact point is located either in the middle of a central aromatic ring (C) or in the center of a C–C bond in the middle (CB) or at the border (B) of the PAHs. Fig. 3 shows the time evolution of the charges for the subfragments presented in the upper panel. It can be seen that the positively charged proton induces electron migrations inside the PAH toward the impact point. Larger charge accumulations are obtained for trajectories colliding a bond than for trajectories passing through an aromatic cycle center (C). Most of the proton-attracted electron density is taken from the nearby fragments which acquire simultaneously positive charges. For the central bond collision (CB), electrons can be attracted from both sides of the bond leading to a larger charge accumulation than for a border bond collision. Once the projectile is gone, electron accumulation becomes energetically unfavorable and, due to electron–electron repulsion, the electronic density flows away from the impact point and the corresponding fragments can even acquire a positive charge. This is the starting point of a period of charge oscillations over the PAH. A similar attraction of electrons through the impact point followed by repulsion has been reported in a very different context of irradiated biomolecules and is known as the ebb-and-flow effect.^209,210^ Interestingly, the size of the PAH seems to have a minor effect on the electron dynamics regarding the intensity of electronic density accumulation, as well as the initial charge dynamics. This is because, on the short timescale, the charge dynamics is very local. This is less true at longer timescales and it can be noticed that high frequency charge oscillations remain clearly visible in the anthracene case whereas in octacene, the charge oscillation becomes more spread over the full PAH, reducing the oscillations intensities.

#### 3.2.2 PAH collision induced fragmentation

Once the electronic system has been excited from a collision, efficient internal conversion is often assumed in PAHs due to conical intersections^73,74^ leading to a redistribution of the absorbed energy over the electronic ground state vibrational modes. The subsequent statistical fragmentation can be described from DFTB-BOMD simulations in the electronic ground state, provided the initial energy distribution is known. In order to simulate the experimental mass spectra of proton-PAH interactions at 100 keV,^69^ a distribution of the deposited energy was estimated from parametrised Monte Carlo simulations (CASPBurn code based on the CASP^211^), and the initial condition for the MD simulations was taken randomly in this distribution. The agreement found between the experimental and theoretical mass spectra suggests the validity of both the energy deposition calculation and the statistical fragmentation hypothesis. In particular, the latter is reinforced by the fact that the main observed and computed fragmentation channels correspond to hydrogen and C_2_H_2_ losses and the absence of single carbon atom fragments, which can be seen as a signature of statistical fragmentation. Gatchell *et al.*^204^ showed from force field and DFTB-MD simulations that in the statistical energy distribution hypothesis, the loss of a single carbon atom does not occur for bare pyrene molecules but that such fragments can be observed for hydrogenated pyrene molecules. The non-statistical fragmentation signatures of PAHs were investigated experimentally and theoretically^82,212^ and occurred to exhibit knockout processes. In order to observe knockout, the collision energy should be in a specific energy range where the nuclear stopping power dominates (the threshold energy for knockout processes was estimated to be about 35 eV for pyrene from MD with classical force field^86^ and observed at 100 eV collision energy for coronene^212^).

### 3.3 Collision induced dissociation of PAH clusters

**Fig. 4:**
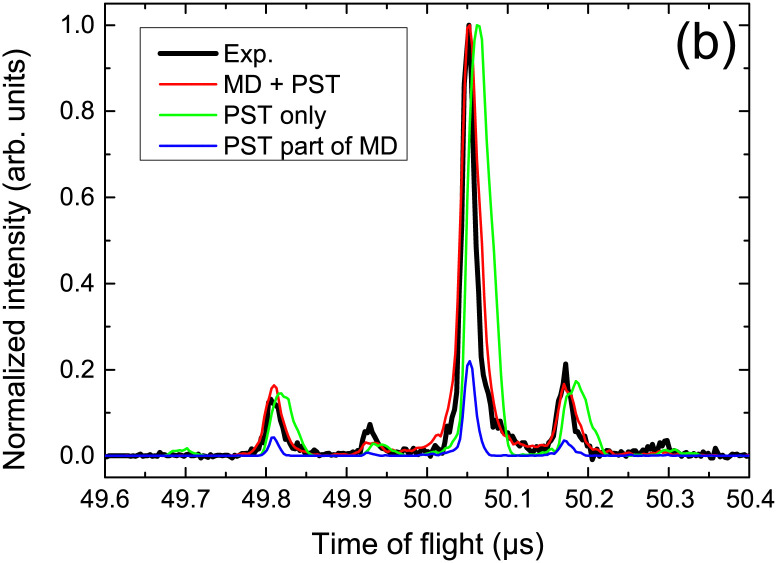
TOF mass spectra for fragment monomers resulting from proton collision-induced dissociation of the cationic pyrene dimer, measured experimentally or simulated assuming a statistical (PST only) *vs.* non-statistical dissociation (DFTB-BOMD + PST). Adapted from ref. 213. with permission from Springer Nature. Copyright 2021.

The collision induced dynamics has also been investigated for PAH clusters. Experimentally, tuning the collision energy of cationic pyrene clusters impacting argon atoms allowed to determine threshold energies for the loss of molecular units,^80,214^ which compared well with the estimated binding energies of these clusters at the DFTB-CI level.^215^ The largest value of about 1.07 eV was obtained for the pyrene dimer cation, slightly decreasing for larger sizes. In the case of cationic dimer dissociations, a deeper analysis of the collision induced dynamics was performed to characterise the dissociation dynamics on the basis of comparisons with experimental time of flight (TOF) mass spectra.^213^ Assuming first that the statistical energy redistribution is fast enough, DFTB-CI binding energies and harmonic vibrational frequencies were used as inputs in a statistical model (Phase Space Theory-PST) to compute evaporation rates and TOF spectra. The latter, however, did not compare well with the experimental ones (see Fig. 4). Explicit MD simulation of the collision and the following 3 ps evidenced that short time dissociations are the dominant processes and that these non-statistical processes are governed by the initial energy deposition conditions, in particular, the impact point and direction of the collision with respect to the intermolecular dissociative breathing mode. After this initial stage, statistical dissociation may occur but with lower intensity. Considering both types of dissociation, the experimental TOF spectra could be recovered as seen in Fig. 4. Monitoring the energy distribution over the various degrees of freedom during the BOMD simulations showed a shorter timescale associated with the energy redistribution between the intramolecular modes of the two units (intramolecular kinetic temperature of the two monomers equalized each to each other in less than 2 ps) whereas the energy transfer is much less efficient between intra and intermolecular modes (sometimes far from being achieved within the simulated 3 ps).

At higher collision energies (keV), the knockout processes within a unit of a PAH cluster have also been investigated. Delaunay *et al.*^71^ identified a series of experimental mass-spectrum peaks as molecular growth fingerprints within the pyrene clusters. Thus intra-cluster growth appears as a possible route for molecular growth towards larger carbonaceous systems. Additionally, BOMD simulations performed with either force field or DFTB potentials confirmed this intra-cluster reactivity.^71^

## 4 Photoinduced dynamics

In this section, we address PAH and PAH cluster evolution following photoabsorption. The choice of the absorbed photon energy is driven both by the available experiments as well as simulating the interstellar UV fluxes conditions.

### 4.1 Electronic excitation and relaxation of photoexcited PAHs

The first steps of photoinduced electronic excitation dynamics can be simulated under the assumption of fixed nuclei. This allowed, for instance, Oviedo, Wong and collaborators^217,218^ to use the RT-TD-DFTB scheme to simulate the electronic response to a laser field excitation of C_60_-dimethylaniline complexes in water or toluene solvents as well as of hydrogenated nanoribbons.

**Fig. 5:**
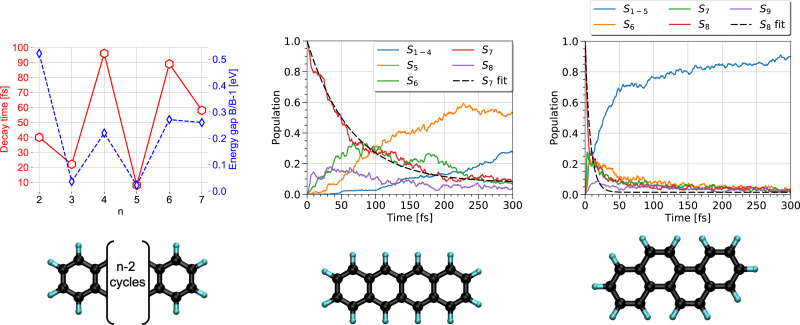
Left: Correlation between the electronic relaxation timescales from the brightest state and energy gap between the latter and the state just below for polyacenes (*n* is the number of aromatic cycles). Time evolution of the state's population for tetracene (middle) and chrysene (right) molecules. Left: Adapted from ref. 191 with permission from the PCCP Owner Societies. Right: Adapted from ref. 216 with acknowledgements of AIP. Copyright 2020.

Once a photon has been absorbed by a PAH molecule, the electron-nuclei dynamics couplings drive the structural evolution of the system and the energy flows between nuclear and electronic degrees of freedom. This can be simulated at the DFTB level by using the TSH strategy (see Section 2.3), to follow the temporal evolution of the population *via* different electronic states and deduce characteristic timescales associated with the diffusion of the energy, initially localized only in the electronic system, towards the vibrational modes. In a first study,^191^ the series of polyacene molecules ranging from naphthalene to heptacene was chosen because their electronic spectra appeared to be reasonably well described with LR-TD-DFTB.^186^ LR-TD-DFTB was used to initiate the TSH-DFTB non-adiabatic simulations from the brightest singlet excited state. These simulations evidenced an alternation in decay times as a function of the number of aromatic cycles, which are typically oscillating between 10 and 100 fs. This alternation shows a correlation with the gap between the initially brightest state and the state lying just below in energy (Fig. 5 left). Using a similar type of simulation, it was shown that while tetracene and chrysene molecules have the same molecular formula, the relaxation is much faster in the case of chrysene (7 fs *vs* 65 fs) (Fig. 5 middle and right).^216^ Let us also mention that, in a photovoltaic context, the relaxation dynamics of electronically excited cycloparaphenylene molecules has also been investigated with DFTB-TSH by Stojanić *et al.*^188^

### 4.2 Photo-induced isomerization/fragmentation of PAHs

**Fig. 6:**
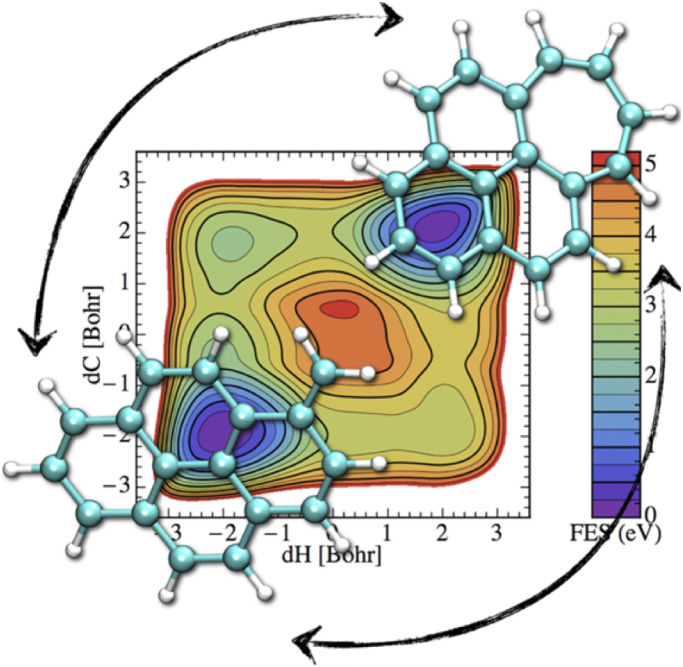
Free energy map for isomerization of the methylene pyrene cation. Reprinted with permission from ref. 220. Copyright 2015 American Chemical Society.

If the electronic relaxation is fast enough and is followed by a redistribution of the absorbed energy over the vibrational modes, prior to major isomerization or fragmentation, the dynamical evolution of a photo-excited PAH can be treated within the “statistical” hypothesis. Note that in such a case, the total absorbed energy, *i.e.* the photon energy, is known, which is a main advantage regarding the modeling, with respect to the simulation of collision induced processes where only an unknown fraction of the collision energy is absorbed. DFT energy calculations can help to identify the plethora of possible competing isomerization processes (hydrogen migration, ring openings, formation of vinilydene groups, *etc.*) and fragmentation (losses of hydrogen or carbonaceous fragments) available at a given energy.^61,90,91,155,219^ At the DFTB level, these competitions can be investigated through metadynamics simulations in order to incorporate entropic effects mapping free energy landscapes. For instance, Fig. 6 represents the free energy map for the transformation of methylene pyrene cation toward a tropylium form ref. 220. It can be seen that two isomerization routes, differing in the ordering of the two involved processes (hydrogen transfer and ring reorganisation), are competitive. Both of them involve two similar and significant free energy barrier heights (3.5–4 eV) showing that the interconversion process would only occur in interstellar clouds under photoactivation. In order to bypass possible chemical intuition bias (predefined path for DFT static exploration, collective variables in metadynamics), DFTB-MD simulations can also be performed providing a blind exploration of all possible isomerization fragmentation processes,^110^ usually identifying hydrogen and C_2_H_2_ losses as major fragmentation channels, in agreement with experimental results. In such simulations, PAHs are provided with vibrational energies much higher than those of the absorbed interstellar photons in order to observe processes within a reasonable computational time, and it is therefore assumed that the competition between the various channels has a low energy dependence.

### 4.3 Photoinduced dissociation of PAH clusters

Several experiments have addressed the photodissociation of PAH clusters. Surprisingly, two of these experiments derived different threshold photon energies for the monomer apparition, namely 0.6^221^ or 1.7 eV.^222^ In addition, these two values also differ from those of the argon collision induced dissociation experiment^214^ which agreed with the theoretical ground state binding energies of 1.07 eV^215^ as already discussed in Section 3.3. This apparent contradiction is related to the initial conditions of the system and the fact that the energy levels of the electronically excited states and their topologies may play a crucial role. Resonance processes of charge and excitations produce several low lying excited states (see Section 2.2 and Fig. 1) which can be tracked using these photodissociation experiments. DFTB-EXCI calculations allowed the interpretation of the different behaviours reported by the different experimental groups.

**Fig. 7:**
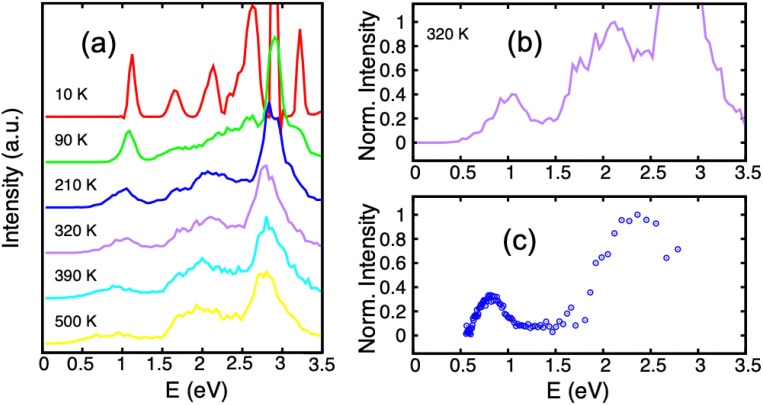
Experimental action spectrum (photodissociation-c) of Py_2_^+^ compared with photoabsorption spectra computed for various temperatures (a) and (b), (DFTB-EXCI). Adapted from ref. 221 with permission from the PCCP Owner Societies. Copyright 2021.

In the first photodissociation experiment,^221^ a population of hot cation dimers is photoexcited. The measured action spectrum, *i.e.* detection of the photodissociation fragments as a function of the absorbed photon energy, shows that photo-dissociation can be induced by photons above ≃0.6 eV (the lower-right curve of Fig. 7). The initial temperature of the system is unknown, but it is hot enough to observe the dissociation of some pyrene dimers without even bringing energy to the system, suggesting that the action spectrum can be compared with a photoabsorption spectrum. From DFTB-EXCI calculations, the first electronic absorption band, which corresponds to an excitation between the two lowest charge resonance states (the two lower curves of Fig. 1(a) and (b)), lies at 1.11 eV when computed for the cationic pyrene dimer optimized geometry. However, when the dimer is hot, it is necessary to take into account all the visited geometrical configurations. This can be understood when looking at the evolution of the two lower potential curves of Fig. 1(a) and (b) as a function of intermolecular distance or twist angle. A parallel tempering Monte Carlo exploration was achieved to sample the visited ground state configurations for temperatures ranging from 10 to 500 K. These distributions were then used to calculate absorption spectra (DFTB-EXCI model), accumulating all single configuration spectral data into histograms at various temperatures. We can see in Fig. 7 (left) that the first absorption band widens and shifts towards low energies when the temperature increases. This is due to the fact that when the temperature increases, the system visits configurations less favourable to charge resonance (*i.e.* smaller overlaps between the HOMOs of the hole accepting monomers). At the highest temperatures, the calculated photoabsorption spectrum approaches the measured action spectrum allowing the estimation of an initial temperature in the 300–400 K range. This is an *a posteriori* confirmation of the hypothesis that the dimers were initially hot, and this initial energy explains why their fragmentation was possible even for photon energies lower than the binding energies.

**Fig. 8:**
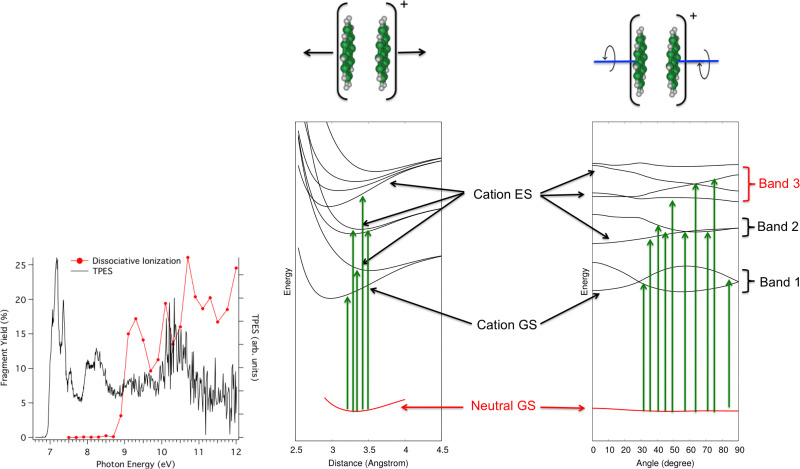
Left: photoionization (black) and photionization + dissociation (red) spectra for Py_2_ recorded at SOLEIL synchrotron apparatus. Right: computed electronic ground states of Py_2_ and Py_2_^+^ and first electronic excited states of Py_2_^+^ according to dissociative breathing mode (left) and non-dissociative twist mode (right). Adapted from ref. 222 with permission from the PCCP Owner Societies. Copyright 2023.

In the second photodissociation experiment^222^ carried out at the SOLEIL Synchrotron facility, neutral pyrene dimers prepared at low temperatures absorb a photon whose energy can be tuned. It is then possible to detect whether the photoabsorption leads to simple ionization (detection of Py_2_^+^, black curve in Fig. 8 – left) or to dissociative ionization (red curve). At first glance, one would expect to observe dissociation when the photon energy is higher than the ionization potential, estimated from the rise of the black curve at about 6.95 eV^223^ plus the binding energy of the pyrene dimer cation (about 1.07 eV from Ar-collision experiment and DFTB-CI calculations^214,215^). The monomer apparition energy is however measured at 8.6 eV, *i.e.* 1.7 eV above the ionization energy. The presence of bands in the photoionization and photodissociation spectra suggests a major role played by the different cation dimer electronic states. The DFTB-EXCI model was used to characterize the topology of the excited PES along two main modes, namely the dissociative breathing mode and the non-dissociative torsion mode (*i.e.* the mode where the molecules twist in opposite directions while keeping the planes parallel). The calculated electronic states PES topologies are shown in Fig. 8. It shows that due to torsion, the states are coupled to each other, which explains the three broad absorption bands. If the absorption takes place (i) in the first band, the pyrene dimer is ionized but does not have enough energy to dissociate, (ii) in the second band, it has enough energy to dissociate but this energy is initially transmitted to a non-dissociative mode. In other words, part of the absorbed energy is given to vibrational energy within the torsion mode because the neutral initial geometry is close to the minimum of the PES curves according to the dissociative breathing mode but not according to the torsion mode. The relocalisation of this energy in the dissociative mode may take place but probably on times larger than those of the experiment and/or of other relaxation channels (like IR emission) and finally (iii) in the third band, the system has enough energy to dissociate and in addition, this energy is initially transmitted to the dissociative breathing mode giving birth to the first dissociation band in red in the experimental spectrum of Fig. 8.

Let us mention other studies addressing the dynamical evolution of electronically excited PAH clusters. The combination of non-adiabatic dynamics with long-range corrected DFTB to investigate the relaxation of excited fluorene oligomers^187^ or to simulate the dynamical evolution of excitons in clusters of tetracene^224^ and perylene di-imides.^225^ The dynamical coupling between local and charge transfer excitons in pentacene clusters was also investigated.^226^ The DFTB-TSH scheme for non-adiabatic dynamics has also been used to simulate excimer formation in the pyrene dimer.^227^

## 5 Conclusion – perspectives

Energy relaxation in PAH molecules and clusters is an important however complex field contributing to understanding the formation and evolution of carbonaceous dust in the interstellar medium. PAH species provide representative and challenging examples of the theory, involving electronic and nuclear relaxation processes at the atomic-, molecular- or nanometer-size scale, running over a variety of time scales. DFTB and its extensions such as TD-DFTB or DFTB-(EX)CI are interesting simulation tools for the electronic and dynamical evolution of molecules and clusters containing up to a few tenths or possibly hundreds of constituent atoms, still retaining a quantum description of electrons and hence relevant to describe chemical reactions and/or electronic processes involving excited states. Thus, excitation of both nuclear and electronic motions can be described, as well as the posterior evolution of the systems, through extensive molecular dynamics simulations including non-adiabatic quantum-classical dynamics.

In this review, we have illustrated a large diversity of mechanisms for energy relaxation in PAH species and how simulations in the DFTB framework can provide support and interpretation for experiments. Examples include both collisional and photon excitation, short time-scales where essentially electronic relaxation is at work, larger time scales where coupled electron-nuclei dynamics occurs, and even longer time-scales, possibly dominated by statistical processes. Significant questions have been or can now be answered such as the collision-induced hydrogenation patterns of PAH, non-adiabatic electronic relaxation or charge dynamics following excitation in linear PAHs and their dependence upon chain size, relaxation through isomerization or dissociative processes, analysis of the contributions of non-statistical *vs* statistical processes, or unraveling the role of specific excitation mechanisms on relaxation in different experiments.

Yet, the field to be explored is vast, as for instance the role of shape (peri-condensed or cata-condensed) and charge (multicharges species) in larger PAHs on the relaxation channels and timescales when the density of electronic states becomes larger. Collisional- or photon-induced interaction of PAH molecules and clusters with other atoms such as oxygen, silicon, nitrogen or iron, consideration of PAH evolution when in contact with various environments such as droplets, clathrates or ice (water, methane) or silicate nanograins provide a rich chemical field to explore. Investigation of bottom-up growth and top-down fragmentation of PAHs or the intra-molecular reactivity and conversion under stellar irradiation will be of great importance to get larger insight and understanding of the carbonaceous content of the interstellar medium. This will obviously make the structural, electronic and dynamical landscape more complex, and it will be interesting to observe how DFTB can respond in more and more challenging size domains and chemical variety. Let us finally mention the growing interest in the environmental topic of oxygenated-PAHs and biomass burning,^228,229^ a research field that has already started to be investigated through force fields^230^ and DFTB potentials.^231,232^

Regarding the description of electronic structures, the DFTB framework seems to offer a relevant frame for medium and larger size molecules and species. Nevertheless, advances still remain to be achieved to provide a general and unified electro-nuclear coupling scheme for all geometrical situations and time-scales. Thus, the problem of fractional charge distribution and ill-behaved charge localization on fragments in dissociative channels, which affect both DFT and DFTB schemes, is still pending (it is related to the self-interaction error, the single determinant support of the density and the use of approximated functionals). It was herein solved in the specific case of singly charged molecular clusters using the DFTB-CI scheme, but it remains to be addressed in a general case. From a molecular dynamics simulation point of view, short-time dynamics (excitation stage and fast relaxation), coupled electronic and nuclear relaxation phases at intermediate times and finally long-time processes often receive relevant but separate dynamical simulation schemes. Alternant use of Ehrenfest dynamics and Tully dynamics during simulations^233^*via* projections of the mean-field state onto the adiabatic excited states may be a way. While classical quantum dynamics is often a relevant tool, some processes (for instance, the presence of conical intersections or the description of coherent non-adiabatic processes) may require consideration of the quantum character of the nuclei, hardly included in simulations with all degrees of nuclear freedom for the systems discussed in this work. Multiple spawning techniques^234^ with nuclear Gaussian functions may offer a promising methodology. Also, light emission following UV irradiation is in competition with other relaxation channels at various time scales and would deserve to be included in the simulations.

## Conflicts of interest

There are no conflicts to declare.

## Supplementary Material
